# Long Noncoding RNA *GAS5*: A New Factor Involved in Bone Diseases

**DOI:** 10.3389/fcell.2021.807419

**Published:** 2022-01-26

**Authors:** Zimo Zhou, Jiahui Chen, Ying Huang, Da Liu, Senxiang Chen, Sen Qin

**Affiliations:** ^1^ Department of Orthopedics, Shengjing Hospital of China Medical University, Shenyang, China; ^2^ Department of Ultrasound, Shengjing Hospital of China Medical University, Shenyang, China

**Keywords:** long noncoding RNA, Gas5, bone disease, osteogenic differentiation, stem cell

## Abstract

Long noncoding RNAs (lncRNAs), as an important type of RNA encoded in the human transcriptome, have shown to regulate different genomic processes in human cells, altering cell type and function. These factors are associated with carcinogenesis, cancer metastasis, bone diseases, and immune system diseases, among other pathologies. Although many lncRNAs are involved in various diseases, the molecular mechanisms through which lncRNAs contribute to regulation of disease are still unclear. The lncRNA growth arrest-specific 5 (*GAS5*) is a key player that we initially found to be associated with regulating cell growth, differentiation, and development. Further work has shown that GAS5 is involved in the occurrence and prognosis of bone diseases, such as osteoporosis, osteosarcoma, and postosteoporotic fracture. In this review, we discuss recent progress on the roles of GAS5 in bone diseases to establish novel targets for the treatment of bone diseases.

## Introduction

As functional RNA molecules, noncoding RNAs (ncRNAs) include four broad categories: (1) microRNAs (miRNAs), (2) long ncRNAs (lncRNAs), (3) circular RNAs, and (4) pseudogenes. ncRNAs were previous thought to lack open reading frames (ORFs) and protein-coding potential. However, more recent studies have shown that ncRNAs are involved in the regulation of gene expression at the transcriptional and post-transcriptional levels (Zhou et al., 2020). Specifically, ncRNAs have roles in epigenetic regulation, chromatin remolding, protein modification, and RNA degradation (Beermann et al., 2016).

**FIGURE 1:**
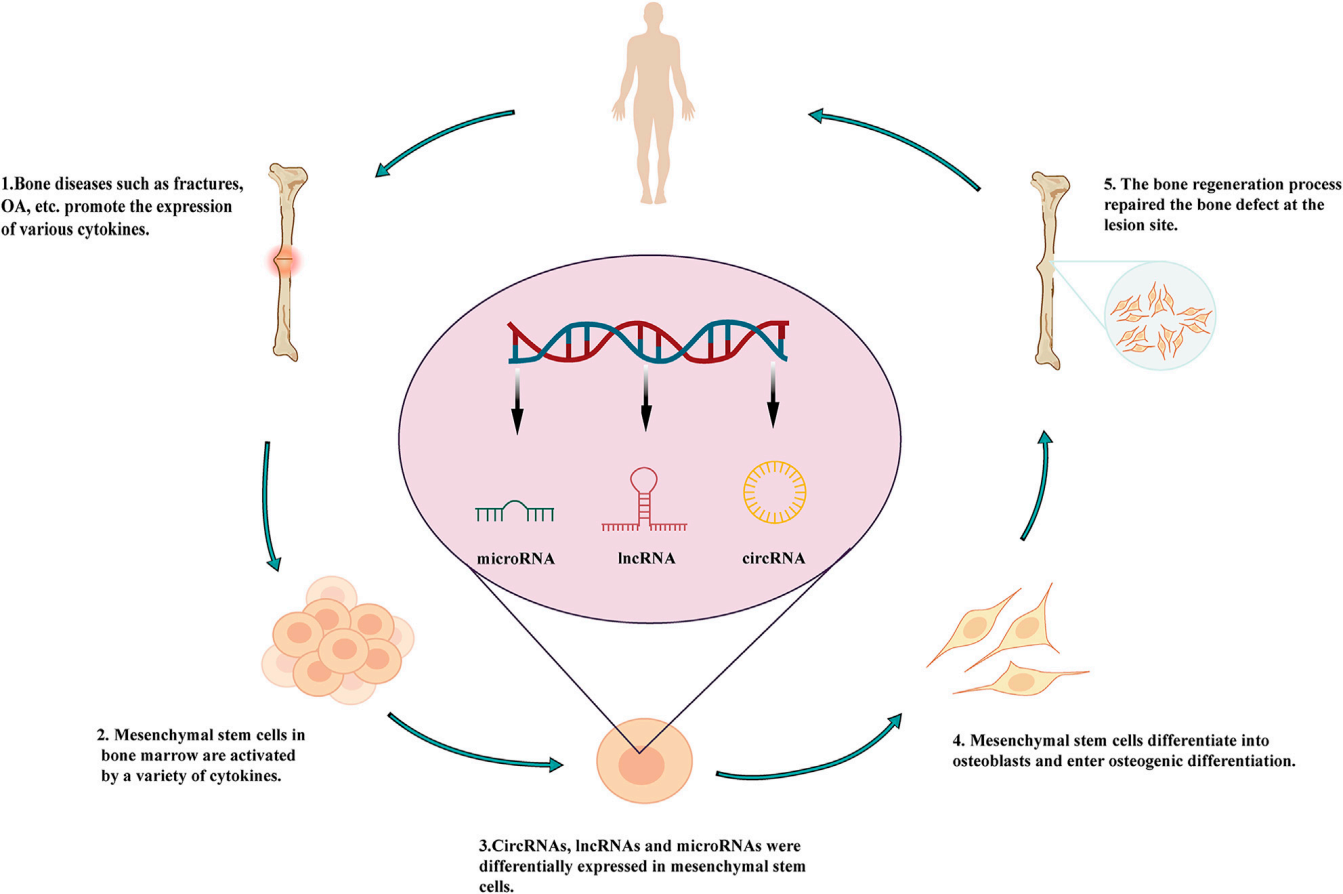
Roles of lncRNAs in the osteogenic differentiation of mesenchymal stem cells and involvement in bone diseases.

LncRNAs are regulatory ncRNAs 200–100,000 nucleotides (nt) in length. These molecules have been shown to play key roles in the occurrence and prognosis of diseases. As polyadenylated byproducts transcribed by RNA polymerase II, lncRNAs were initially thought to be a type of transcriptional noise without any real functions (Peng et al., 2018). However, advances in RNA-binding protein immunoprecipitation (Fuentes-Iglesias et al., 2020), RNA pull-down assays, and other sequencing technologies (Takahashi et al., 2012; Heather and Chain, 2016) have shown that lncRNAs regulate many complex processes in different diseases (Chen et al., 2020; Ghafouri-Fard et al., 2020; Zhang, Guo et al., 2020 Zhao, Pathak et al., 2020), including bone diseases such as osteoporosis, osteoarthritis (OA), ankylosing spondylitis (AS), osteosarcoma, and bone fracture (Figure 1). Furthermore, several studies have confirmed the involvement of lncRNAs in bone diseases (Li, Yun et al., 2020; Chatterjee et al., 2020; Hong et al., 2020; Kushlinskii et al., 2020). For example, Núnêz et al. (2000) showed that *H19* expression is downregulated in the myeloproliferative disease and that *H19* regulates insulin-like growth factor 2 in stem cells. Moreover, lncRNAs have also been shown to regulate the osteogenic differentiation of stem cells in bone diseases.

The lncRNA growth arrest-specific 5 (*GAS5*) was initially identified in NIH 3T3 cells using subtraction hybridization. In human cells, *GAS5* was shown to be transcribed from a small ORF on chromosome 1q25.1 (Schneider et al., 1988; Isin et al., 2014). Moreover, *GAS5* was subsequently shown to interact with the DNA binding domain of glucocorticoid receptors to suppress multiple anti-apoptotic genes, thereby enhancing the sensitivity of cells to apoptotic signals (Kino et al., 2010). Researchers are currently interested in evaluating the roles of *GAS5* expression in bone diseases. For example, Visconti et al. (2020) found that *GAS5* expression levels were significantly altered in the blood of patients with osteoporosis compared with that in healthy individuals. In another study, *GAS5* was shown to be differentially expressed in patients with osteoporosis (Centofanti et al., 2020). However, few reports have evaluated the roles of *GAS5* in bone diseases.

In this review, we discuss the expression of *GAS5* in bone diseases and the mechanisms through which this lncRNA may contribute to the pathological state. Furthermore, we discuss problems with studies of *GAS5* function in order to facilitate further research of this important lncRNA in bone diseases.

## Regulatory Mechanisms of *GAS5*

The *GAS5* gene was first described by Schneider et al. (1988). Subsequently, Coccia et al. (1992) showed that the gene encoding *GAS5* has a 5′-terminal oligopyrimidine belonging to an upstream oligopyrimidine tract sequence. Since the transcription product of the *GAS5* gene can accumulate in growth-arrested cells, the lncRNA is called growth arrest-specific 5 (*GAS5*). The gene is located on chromosome 1q25 and is 630 nt in length, with 12 exons and a short ORF. When the exons are transcribed, the product can be spliced into two possible mature lncRNAs, *GAS5a* and *GAS5b*, through alternate splicing. Furthermore, during *GAS5* transcription, there are many different patterns of alternate splicing. Notably, the activity of *GAS5* may be associated with the introns, which encode small nucleolar RNAs (Shi et al., 2013).

In T cells, serum starvation and rapamycin treatment increase GAS5 expression via a mechanism involving the mammalian target of rapamycin pathway (Mourtada-Maarabouni et al., 2010). Kino et al. (2010) found that *GAS5* increases sensitivity to apoptosis by inhibiting the anti-apoptotic activities of glucocorticoids. During this mechanism, *GAS5*, acting as a starvation- or growth arrest-linked riborepressor for the glucocorticoid receptor (GR), can bind to the DNA-binding domain, inhibiting the interaction between target DNA and its receptors.

In recent studies, *GAS5* has been shown to be associated with tumors, functioning as a tumor suppressor, with roles in tumor occurrence, metastasis, necrosis, and prognosis (Zhao, Zheng et al., 2020). Moreover, in a screening of GAS5 expression in different breast cell lines, researchers showed that *GAS5* transcriptional products with different exon/intron splicing combinations stimulate apoptosis through various cellular signaling pathways (Mourtada-Maarabouni et al., 2009).

### *GAS5* as a Modulator of the Nucleoprotein Complex

In placental mammals, chromatin silencing machinery, which is associated with the lncRNA/protein complex, has important biological roles, turning off the transcription of many genes on the inactive X chromosome (Penny et al., 1996). Biological techniques derived from these findings are widely used in various research fields. Importantly, ribosomes may bind to smORFs (small open reading frames) in one lncRNA without translating the ORF via ribosome “sponging”, thereby inhibiting translation (Goustin et al., 2019).

### Regulation of Apoptosis by *GAS5* as a Glucocorticoid Receptor Mimic

*GAS5* can fold into RNA secondary structures to compete with the GR for binding onto its target (Kino et al., 2010). Moreover, *GAS5*, as a competitive inhibitor of GR, can regulate cell apoptosis. In this mechanism, GR first binds to the GR ligand binding domain in the cytoplasm and then translocates into the nucleus to bind to specific GR DNA binding sequences to regulate the transcription of target genes. Studies are currently underway to use the GR element mimic to assess the lncRNA/protein binding domain in *GAS5*.

### Interaction of *GAS5* Transcripts with miRNAs

LncRNAs can serve as competing endogenous RNAs (ceRNAs), sponging the target miRNAs through homology similar to the miRNA/mRNA interaction, thereby regulating the expression and function of miRNAs (Liu et al., 2018). Several studies have shown that *GAS5* can associate with miRNAs in many disease states. For example, *GAS5* contributes to the development of breast cancer via a ceRNA-dependent mechanism. Like a sponge, *GAS5* can directly associate with the binding site in *miRNA-23a*, inactivating the *miRNA-23a* mimic and counteracting the negative effects of the *miRNA-23a* mimic on ATG3 to promote autophagy in breast cancer cells (Gu et al., 2018b). In other studies of breast cancer, different miRNAs, including *miR-221/222*, *miR-196a-5p*, *miR-378a-5p*/*SUFU*, and *miR-21*, have been shown to sponge with *GAS5* (Li et al., 2016; Mei et al., 2017; Xue et al., 2017; Gu et al., 2018a; Li et al., 2018; Toraih et al., 2018; Zhao et al., 2018; Dong et al., 2019; Yang and Jiang, 2019; Zong et al., 2019; Wang, Ke et al., 2020; Zheng et al., 2020). These interactions have also been confirmed in other cancers (Table 1). However, the specific signaling pathways involved are still unclear, and further validation studies are needed to fully elucidate the roles of *GAS5* in mediating miRNAs in various disease states.

**TABLE 1:** The roles of different microRNAs associated with *GAS5*.

MicroRNA	Downstream target	Tumor or cell type	Effect	References
*miRNA-23a*	ATG3	Breast cancer	Sponge	Gu et al. (2018b)
				Li Zhao et al. (2018)
*miR-196a-5p*	FOXO1/PI3K/AKT			
				Zheng et al. (2020)
*miR-378a-5p*	SUFU			
				Zong et al. (2019)
*miR-221/222*	Unknown			
				Li et al. (2016)
*miR-21*	Unknown			
*miR-205*	PTEN	Non-small cell lung cancer	Sponge	Dong et al. (2019)
*miR-135b*	Unknown	Cell lung		Xue et al. (2017)
		Cancer		Mei et al.2017
*miR-23a*	Unknown			
*miR-21*	PTEN	Hepatocellular carcinoma	RISC sponge*GAS5* and miRNA coregulated	Wang, Ke et al. 2020
*miR-135b*	RECK			Yang et al. (2019)
*miRNA-34*	Different pathways			Toraih et al. (2018)
*miR-196a-5p*	HOXA5	Ovarian Cancer	Sponge	Zhao et al. (2018)

MSCs: mesenchymal stem cells; VSMCs: vascular smooth muscle cells; BMSCs: bone marrow mesenchymal stem cells; PDLSCs: periodontal ligament stem cells; ceRNA: competing endogenous RNA.

### Other Possible Regulatory Mechanisms of *GAS5*

*GAS5* may also mediate disease states through various other mechanisms. In breast cancer, Li et al. (2019) found that *GAS5* upregulation promotes the chemosensitivity and apoptosis of triple-negative breast cancer (TNBC) cells. Methylation of CpG islands in the promoter region of *GAS5* was identified in TNBC tissues, indicating that aberrant methylation affects the biological activity of *GAS5*. Furthermore, upregulation of *GAS5* via suppression of methylation was shown to accelerate apoptosis in TNBC cells.

In another study, *GAS5* was found to be associated with the promoter element of the insulin receptor, altering its expression in patients with type 2 diabetes mellitus. Moreover, the stability of *GAS5* is regulated by degradation rather than transcription. Indeed, limiting the degradation of *GAS5* by blocking its interaction with up-frameshift mutant (UPF1) increases *GAS5* expression and glucose uptake in adipocytes from patients with diabetes (Goustin et al., 2019; Shi et al., 2019). These findings suggest that *GAS5* may bind to genomic DNA to regulate various cellular processes.

YES-associated protein (YAP) activation plays key roles in cancer development by regulating target gene expression via formation of complexes with multiple transcription factors. Phosphorylation of a different locus in YAP, an important downstream locus of the Hippo pathway, can regulate nuclear and cytoplasmic localization (Moon et al., 2017). The N6-methyladenosine (m6A) modification is introduced by the m6A methyltransferase complex. Ni et al. (2019) showed that *GAS5* can directly interact with the WW domain of YAP to promote the translocation of YAP from the nucleus to the cytoplasm and enhance the phosphorylation and ubiquitin-mediated degradation of YAP. This mechanism results in decreased expression of the YAP-mediated transcription product of YTHDF3, which reversibly and selectively binds m6A-methylated *GAS5* to trigger its decay, generating a negative feedback loop.

Accordingly, *GAS5* may regulate different cellular metabolic pathways and processes to promote disease development. Further studies are needed to identify new *GAS5-*related treatment targets based on these mechanisms. Moreover, validation studies are needed to determine whether these mechanisms are truly involved in disease onset and progression.

## Roles of *GAS5* in the Osteogenic Differentiation of Different Types of Stem Cells

**FIGURE 2:**
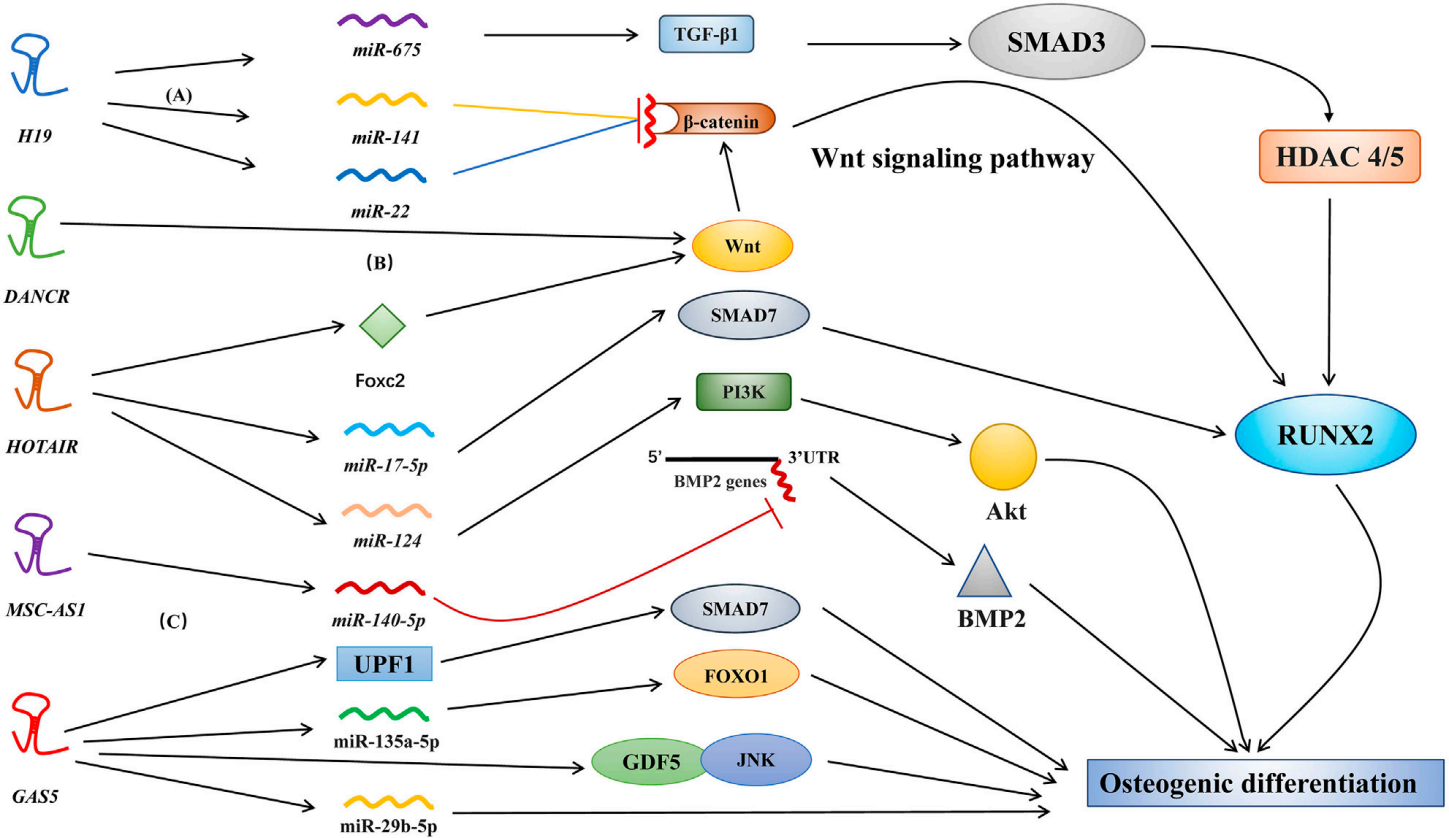
Different molecular pathways of lncRNAs in osteogenic differentiation. Various lncRNAs, such as *H19*, *MALAT1*, *HOTAIR*, and *MSC-AS1*, regulate osteogenic differentiation via targeting miRNAs. **(A)** *H19* actively regulates the Wnt signaling pathway by inhibiting the binding of *miR-141* and *miR-22* to β-catenin (Gong et al., 2018). **(B)** *DANCR* regulates osteogenic differentiation via targeting the Wnt signaling pathway. However, the specific factors regulated by *DANCR* have not yet been identified (Jiang et al., 2019). **(C)** *MSC-AS1* suppressed *miR-140-5p* expression to upregulate *BMP2* via inhibition of *miR-140-5p* binding to the 3′ UTR of the *BMP2* gene; this promotes osteogenic differentiation (Zhang et al., 2018).

In several types of stem cells, osteogenic differentiation can promote bone regeneration and bone healing. Osteoblasts and osteoclasts are key cells involved in osteogenic differentiation and bone turnover. Osteoblasts are differentiated from bone-derived stem cells, such as mesenchymal stem cells (MSCs), periodontal ligament stem cells (PDLSCs), and human dental pulp stem cells (Zhao, Tu et al., 2020; Kichenbrand et al., 2020; Tian et al., 2020). Various molecular pathways, including lncRNAs, regulate the complex osteogenic differentiation process. For example, in osteogenic differentiation, lncRNAs have been shown to modulate bone morphogenetic protein (BMP) (Zhang, Du et al., 2019), the WNT/β-catenin/RUNX2 pathway (Shen et al., 2019; Zhou et al., 2019), and the transforming growth factor-β (TGF-β)/Smad3 pathway (Huang et al., 2015) (Figure 2) through three major mechanisms, including epigenetic modifications, miRNA sponges or precursors, and direct effects on molecular targets.

With advancements in genetic technologies, epigenetic modifications, including DNA methylation, histone modification, and RNA modulation have been analyzed in yeast, plants, and mammals. The important roles of lncRNAs in epigenetic, transcriptional, and post-transcriptional gene regulation, such as X-chromosome inactivation, histone modification, imprinting, transcriptional interference, and nuclear and cytoplasmic trafficking, have been identified in all stages of osteogenic differentiation of stem cells (Martianov et al., 2007; Rinn et al., 2007; Skvortsova et al., 2018).

Furthermore, researchers have recently started evaluating lncRNA/miRNA sponges in osteogenic differentiation. For example, metastasis-associated lung adenocarcinoma transcript 1 (*MALAT1*) can sponge *miR-30* in adipose-derived MSCs (Yi et al., 2019), *miR-124* in MSCs (Zhang, Pathak. et al., 2020), and *miR-204* in human aortic valve interstitial cells (Huang et al., 2015; Jiang et al., 2017; Wang et al., 2017; Wei et al., 2017; Xiao et al., 2017; Xiao et al., 2017; Wang, Zeng et al., 2018; Wang, Liu et al., 2018; Gao et al., 2018; Wu et al., 2018; Liu et al., 2019; Yang et al., 2019; Yi et al., 2019; Wang, Xiao et al., 2020; Zhang, Wang et al., 2020; Pan et al., 2020).

As an important regulatory factor, *GAS5* is also involved in osteogenic differentiation. For example, *GAS5* can regulate MSCs through various mechanisms and promote or inhibit the occurrence and development of bone diseases. Here, we summarize the potential mechanisms of action of *GAS5* in various types of stem cells and discuss the regulatory effects involved in these mechanisms (Table 2).

**TABLE 2:** Regulation and expression level of *GAS5* in different MSCs.

Molecular functions	Expression level of *GAS5* in osteoporosis	Targets	Cell model	Regulatory effect	References
N/A	Overexpression	N/A	Human blood	N/A	Goustin et al. (2019)
N/A	Low expression	N/A	Human MSCs	N/A	Visconti et al. (2020)
Nonsense-mediates mRNA decay	Low expression	UPF1	Human MSCs	Promotion	Li, Yun et al. 2020
ceRNA	Low expression	*miRNA-498*/RUNX2	Human MSCs	Inhibition	Feng et al.2019
	Low expression	*miR-26b-5p*/PTEN	Human VSMCs	Inhibition	Leopold et al. (2015)
	Low expression	*miR-135a-5p*/FOXO1	Mouse BMSCs	Promotion	Wang et al. (2019)
Phosphorylation	Low expression	GDF5	Human PDLSCs	Promotion	Yang et al. (2020)

MSCs: mesenchymal stem cells; VSMCs: vascular smooth muscle cells; BMSCs: bone marrow mesenchymal stem cells; PDLSCs: periodontal ligament stem cells; ceRNA: competing endogenous RNA.

### Human Mesenchymal Stem Cells

MSCs are multipotent stem cells that can differentiate into a broad range of cell types, including osteoblasts, adipocytes, chondrocytes, tendon cells, and myocytes. MSCs have important potential in bone regeneration and tissue repair in bone, adipose, cartilage, and muscle tissues (Yang et al., 2018; Feng et al., 2019). Bone marrow MSCs (BMSCs) and adipose tissue-derived stromal stem cells are two types of MSCs that show high proliferative capacity and potential regenerative properties (Yang et al., 2018).

The regulation of different lncRNAs in MSCs has been widely reported. For example, the lncRNA *H19* is involved in the osteogenic differentiation of MSCs, modulating various regulatory factors and pathways, such as the Wnt/β-catenin pathway and the TGF-β1/Smad3/histone deacetylase (HDAC) signaling pathway (Huang et al., 2015; Zhou et al., 2019). However, the molecular mechanisms of *GAS5* in MSCs are unclear. In one study, Li et al. (2020) found that *GAS5* overexpression significantly enhanced alkaline phosphatase activity and promoted the osteogenic differentiation of human MSCs by interacting with UPF1 to degrade *SMAD7* mRNA. These findings further established a novel pathway through which lncRNAs regulate nonsense-mediated mRNA decay, a highly conserved mechanism widely present in eukaryotes. In addition, *GAS5* has been shown to modulate the *miR-135a-5p*/FOXO1 pathway by functioning as a ceRNA (Wang et al., 2019). The *GAS5*/SMAD7 axis and *GAS5/miR-135a-5p*/FOXO1 axis in MSCs are both involved in the development and prognosis of osteoporosis and will be described in greater detail in Section 4.1.

### Human Periodontal Ligament Stem Cells

Human periodontal ligament stem cells (hPDLSCs), as potential seed cells in bone engineering tissue, may contribute to alveolar bone regeneration (Jia et al., 2019). Knockdown of *GAS5* inhibits the osteogenic differentiation of hPDLSCs. In contrast, overexpression of *GAS5* promotes osteogenic differentiation. Yang et al. (2020) also found that *GAS5* overexpression increases the level of growth differentiation factor 5 and accelerates the phosphorylation of JNK and p38 in hPDLSCs. However, the specific molecular mechanisms through which *GAS5* functions in hPDLSCs remain unclear.

### Human Vascular Smooth Muscle Cells

Vascular calcification plays important roles in the occurrence and development of cardiovascular disease and chronic kidney disease. This mechanism may be associated with the osteogenic differentiation of human vascular smooth muscle cells (hVSMCs) (Leopold, 2015). In one study, Chang et al. (2020) found that *GAS5* was significantly downregulated in hVSMCs. Subsequent research showed that *GAS5* overexpression positively regulates phosphatase and tensin homolog levels through *miR-26b-5p* sponging, thereby inhibiting osteogenic differentiation by inducing high levels of phosphorus in hVSMCs.

## Roles of *GAS5* in Bone Diseases

Bone diseases were previously thought to be disorders of the absorption and release of calcium and phosphorus. However, recent studies have demonstrated the involvement of bone-derived cells and related molecules in bone diseases. Moreover, lncRNAs can also regulate the development of bone diseases. As a key factor in osteogenic differentiation, *H19* has potential biological roles in bone diseases, such as osteoporosis (Li, Zhao et al., 2018; Xie et al., 2019), osteoarthritis (Steck et al., 2012; Chen et al., 2016), and fracture (Zhou, Yu et al., 2018; Li Yun et al., 2020). However, the roles of *GAS5* in bone diseases are still unclear, and most studies of *GAS5* in bone diseases have been limited to studies of osteoporosis and osteosarcoma.

### Osteoporosis

Patients who have osteoporosis suffer from a higher risk of fractures. In older women in particular, the rate of fractures due to osteoporosis is greatly increased because of changes in estrogen levels (Väänänen and Härkönen, 1996). In the past, patients who had bone fractures often required surgery. In particular, patients with hip fractures often required hip replacements, resulting in poor quality of life, a dependent living situation, and an increased risk of death (Cummings and Melton, 2002). However, many recent clinical studies have focused on prevention of future fractures in patients with postmenopausal osteoporosis (Black and Rosen, 2016). Age-related osteoporosis involves lack of new bone formation and the accumulation of fat in the bone marrow compartment, which can be associated with reduced osteoblast number and impaired differentiation of MSCs into adipocytes (Li, Xiao et al., 2018).

LncRNAs, as key factors regulating osteogenic differentiation, may have potential applications in the treatment of osteoporosis. For example, the novel lncRNA *Bmncr* modulates the age-related osteogenic niche (Li, Xiao et al., 2018). Indeed, *Bmncr* regulates the osteogenic niche of BMSCs by maintaining the expression of the extracellular matrix protein fibromodulin and the activation of the BMP2 pathway. *Bmncr* can also act as a scaffold to promote interaction of TAZ and ABL, thereby facilitating the assembly of the TAZ and RUNX2/PPARG transcriptional complex, promoting osteogenesis, and inhibiting adipogenesis.

*GAS5* has also been shown to have key functions in osteoporosis. The *GAS5* expression level is significantly downregulated in patients with osteoporosis (Centofanti et al., 2020). Moreover, Visconti et al. (2020) showed that *GAS5* may have great value as a putative prognostic biomarker in patients with osteoporosis and osteoporosis-related fractures. In addition to its roles in the *GAS5*/SMAD7 and *GAS5/miR-135a-5p*/FOXO1 axes, *GAS5* also had other regulatory effects in osteoporosis. For example, quantitative real-time polymerase chain reaction showed that patients with osteoporosis exhibit high levels of *miR-498* and low levels of *GAS5* and *RUNX2*. In further experiments, *GAS5* was shown to significantly regulate *RUNX5* expression via *miR-498* (Feng et al., 2019). In other studies, *GAS5* expression had altered in patients with osteoporosis (Centofanti et al., 2020; Visconti et al., 2020).

Overall, although many researchers are currently evaluating the mechanisms through which *GAS5* mediates osteoporosis, information is still limited, and more robust studies are needed to confirm these findings. Furthermore, whether and how *GAS5* regulates the same pathway through different miRNAs and the specific molecules that bind with *GAS5* to modulate crosstalk among different pathways are still being investigated.

### Osteosarcoma

Osteosarcoma is a common type of primary malignant tumor occurring primarily in children and adolescents. Osteosarcoma often arises in the long bones of the limbs, such as the femur, tibia, and humerus, near the metaphyseal growth plate (Zambo and Veselý, 2014). Surgery is still the major treatment strategy for patients with osteosarcoma; however, the survival rate of patients who undergo surgery alone is low. In recent decades, the combination of surgical treatment and chemotherapy has resulted in significantly increased survival rates (Kansara et al., 2014). However, metastasis, recurrence, and drug resistance seriously affect patient prognosis (Bielack et al., 2002; Ritter and Bielack, 2010). Therefore, analysis of the molecular mechanisms of osteosarcoma development may establish novel biomarkers for the treatment of this disease (Rossi et al., 2019; Czarnecka et al., 2020).

LncRNAs have also been shown to have important roles in osteosarcoma via regulation of multiple pathways *in vivo* and *in vitro* (Yan et al., 2018; Zhang et al., 2018; Fu et al., 2019; Zheng et al., 2019). Moreover, various lncRNAs alter tumorigenesis and metastasis by upregulation or downregulation of their targets. For example, the lncRNA *SNHG12* promotes the development of osteosarcoma by upregulating Notch2 via sponging *miR-195-5p* (Zhou, Zhang et al., 2018).

Importantly, *GAS5*, which acts as a sponge for *miR-221* (an miRNA that downregulates ARHI), exhibits antitumor effects and modulates the epithelial-mesenchymal transition by enhancing ARHI expression (Ye et al., 2017). As an alternate mechanism, HDAC1/2, which forms a heterodimer, can bind to the transcriptional complex composed of IRF1 and C-terminal binding protein 1 (CtBP1), called the CtBP1-HDAC1/2-IRF1 transcriptional complex; this complex targets the *GAS5* promoter and suppresses *GAS5* expression. In another study, downstream targets of *GAS5*, including tumor-suppressor genes (e.g., *TP53*, *Bax*, and *Bim*) as well as oncogenes (e.g., *TGFB*, *DDB2*, and *ROS1*), were identified (Zhang, Hu et al., 2019), further highlighting the roles of *GAS5* in osteosarcoma.

## Conclusion

In recent studies, researchers have shown that lncRNAs can be used as biomarkers for the prognosis, prevention, and treatment of various diseases, including bone-related diseases. Although the complex mechanisms through which lncRNAs contribute to diseases have not been fully elucidated, studies have demonstrated their potential applications in clinical practice. *GAS5*, a key regulatory molecule, can suppress cell growth to modulate the disease state. In normal cells, *GAS5’s* expression is low; however, in tumor tissues and other diseased tissues, *GAS5* is upregulated. With the development of biotechnologies, such as sequencing-based methods (Qian et al., 2019), researchers will be able to elucidate additional signaling pathways and targets. Moreover, additional work is still needed to confirm the findings reported in the current body of literature. For example, *in vivo* experiments are required to validate the roles of *GAS5* demonstrated in basic cellular studies. In addition, many of the details of *GAS5* activity and mechanisms are still unclear. Furthermore, whether and how *GAS5* regulates relevant mechanisms in different bone-derived stem cells, whether and how *GAS5* regulates the same pathway mediated by different miRNAs, which molecules harbor *GAS5* binding sites to facilitate crosstalk among different pathways, and whether *GAS5* has roles in other bone diseases, such as OA and AS have not yet been clarified. Therefore, intensive discussions are required to decide how to dissect the roles of *GAS5* in particular systems, such as bone tissue. Finally, further studies are necessary to translate basic research into clinical practice.
